# An allometric large-scale analysis of the observed performance limits and sex differences in elite powerlifting

**DOI:** 10.3389/fphys.2026.1847605

**Published:** 2026-06-24

**Authors:** Simone Montenegro, Pamela Wicker, Lars Donath

**Affiliations:** 1 Department of Applied Exercise Science, German Sport University Cologne, Cologne, Germany; 2 Department of Medicine and Aging Sciences, Università degli Studi “G. d’Annunzio” Chieti-Pescara, Chieti, Italy; 3 Department of Neurosciences, Biomedicine and Movement, University of Verona, Verona, Italy; 4 Department of Sports Science, Bielefeld University, Bielefeld, Germany

**Keywords:** allometric scaling, biological limits, performance normalization, powerlifting, sex dimorphism

## Abstract

**Background:**

The objective normalization of athletic performance across diverse body masses remains one of the most persistent methodological challenges in strength and conditioning research. Historically, this normalization has been governed by the theoretical square-cube law (scaling exponent b = 0.67) or, more recently in strength sports, by highly parameterized polynomial algorithms such as the Wilks and International Powerlifting Federation General Load (IPF GL) formulas. This study challenges both geometric scaling and polynomial overfitting by deriving empirical allometric exponents from a tiered stratification design.

**Methods:**

A large-scale, highly curated dataset of powerlifters competing under performance-enhancing drug testing protocols was analyzed (N = 457,471). A stratified log-log regression model was implemented, progressively restricted to the absolute Top 20 all-time athletes per weight category (N = 1,833) to identify shifting scaling laws. Comparative statistical equivalence and agreement with the IPF GL formula were evaluated using Williams' t-test and Bland-Altman analysis.

**Results:**

The research identified a systemic phenomenon termed "allometric decay". As athletes approach absolute physiological limits, the scaling exponent meaningfully deviates from 0.67, decaying from general population baselines (males: b = 0.55; females: b = 0.50) down to 0.47 for elite males and 0.41 for elite females, at the Global All-Time Top 20 level. Despite its mathematical simplicity, the empirical allometric model achieved statistical equivalence with the complex IPF GL formula in mass-independence for elite males (Williams' t = -0.72, p = 0.47, Cohen's q = 0.001) and demonstrated extremely high-ranking agreement (mean bias = 0.00; 95% limits of agreement = ± 0.20 Z-scores).

**Conclusion:**

This significant decay indicates that the observed cost of acquiring functional muscle mass exhibits progressive diminishing returns at the elite level. Consequently, current polynomial scoring systems appear to be mathematically overfitting data to compensate for these underlying physiological ceilings. This study proposes a return to sex-specific empirical allometry, advocating for the adoption of these derived exponents as a more parsimonious, transparent, and biologically grounded method for cross-category strength comparison.

## 1 Introduction

The endeavor to compare strength performance across body mass categories is a secondary but meaningful pursuit in strength sports, particularly for “Best Lifter” determinations, while primary competitive outcomes are decided by absolute loads lifted within a given weight class. The mathematical models utilized to achieve this cross-category comparison have nonetheless been subjects of continuous debate (Batterham and George, 1997). The core of this debate revolves around the tension between theoretical physics and biological reality. Since the early biomechanical postulates formalized in Galileo Galilei’s seventeenth century theses, the square-cube law has served as the baseline heuristic approach for scaling biological functions. The law posits that the maximal force a muscle can produce is directly proportional to its physiological cross-sectional area, which scales with the square of linear dimensions, whereas body mass scales with the cube of those dimensions. Under the assumption of perfect anatomical isometry, maximum strength should, therefore, scale to body mass raised to the power of 0.67 (Nevill and Holder, 1995). The geometric similarity assumption underlying this scaling law has itself been questioned in the context of athletic populations (Nevill et al., 2004). It is important to note that intra-category competition is resolved by absolute loads lifted; allometric normalization becomes relevant specifically when performances are compared across weight categories, such as in the determination of “Best Lifter” awards.

However, human beings are not perfectly built structures. As the body mass increases, anthropometric proportions, body composition, and neuromuscular efficiency undergo significant nonlinear alterations. In the context of elite powerlifting, athletes in heavier weight categories invariably exhibit different morphological characteristics compared to their lighter counterparts. Specifically, the accumulation of body mass eventually collides with the physiological ceiling of the Fat-Free Mass Index (FFMI). Once an athlete approaches this hypertrophic limit, any further increase in total body mass is disproportionately composed of adipose tissue and extracellular fluid. While this additional mass may provide passive structural stability and favorable joint angles in certain compressive movements, it does not contribute to active force production, effectively functioning as dead weight (Kouri et al., 1995).

Despite the well-documented failure of geometric scaling to account for these biological ceiling effects, international federations have historically avoided simple allometric modeling in favor of complex polynomial regressions. Scoring systems such as the Wilks formula, the Dynamic Objective Team Scoring (DOTS) system, and the currently utilized International Powerlifting Federation General Load (IPF GL) formula attempt to normalize performance by fitting third-degree, fourth-degree, or even fifth-degree polynomials to historical competitive data (Sinclair, 1985; Vanderburgh and Batterham, 1999). While these algorithms successfully generate a smooth curve that attempts to equalize the probability of winning across all weight classes, they suffer from profound epistemological and practical flaws. First, they operate as mathematical “black boxes” lacking any explanatory physiological mechanism. Second, highly parameterized polynomials are inherently susceptible to demographic bias and overfitting; they describe the historical distribution of a specific dataset rather than uncovering the underlying biological laws governing human strength (Jaric, 2002). Indeed, Arandjelović (2013) has argued that regression-based normalization in strength sports is inherently biased by the demographic composition of the reference population, a concern that directly motivates the present allometric approach. The allometric framework applied here builds on Cleather (2006), who provided the foundational methodological reference for allometric scaling in powerlifting specifically.

This investigation hypothesizes that the perceived necessity for complex polynomial formulas arises from a fundamental misunderstanding of allometry in sports science. The allometric exponent is neither a static universal constant, nor should it be artificially curved by algorithms. Instead, it is a dynamic parameter that systematically decays as the athletic population approaches the observed performance limits of the sport. A recent analysis of the same OpenPowerlifting database by Ferland et al. (2020) benchmarked existing polynomial formulas against one another; the present study extends that work by deriving empirical allometric exponents through a tiered stratification design rather than comparing pre-existing scoring systems. By isolating the absolute highest performers in the history of powerlifting who compete under performance-enhancing drug testing protocols, this study aims to derive the pure, empirical allometric slopes for both male and female athletes, thereby providing a transparent and empirically grounded alternative to current normalization algorithms. It should be acknowledged that drug-testing protocols, including those enforced by the IPF, carry a non-zero false-negative rate; the present dataset therefore cannot guarantee a fully drug-free population. Ultimately, establishing these empirically observed performance boundaries is crucial for replacing arbitrary mathematical scoring with an equitable, evidence-based framework that allows federations and practitioners to accurately evaluate human strength across the entire body mass spectrum.

## 2 Materials and methods

### 2.1 Procedures and data filtration

To accurately quantify the biological limits of human strength, it was imperative to construct a dataset isolated from confounding variables such as performance-enhancing drugs, supportive equipment, and sub-maximal genetic expression. Data for this retrospective observational study were extracted from the Open Powerlifting project database (accessed in March 2026) (Open Powerlifting, 2026), the most comprehensive public repository of competitive results globally. The initial dataset underwent a multi-stage filtration process to ensure maximum physiological validity. Inclusion was strictly limited to the “Raw” division, thereby excluding athletes utilizing knee wraps or supportive suits, as the elastic deformation of such equipment artificially alters natural biomechanics and allometric scaling. Furthermore, the sample was restricted to federations enforcing rigorous out-of-competition drug testing protocols, primarily the International Powerlifting Federation (IPF) and its national affiliates. To mitigate the influence of neuromuscular and musculoskeletal aging, the age range was bounded between 20 and 35 years, capturing the recognized window of peak physiological strength. Finally, only athletes who successfully recorded a valid total across the squat, bench press, and deadlift in a single sanctioned meet were retained for analysis.

The IPF has revised its official weight classes on multiple occasions throughout its history. In the present analysis, athletes were classified according to the weight class under which their competitive result was officially recorded at the time of competition. Stratification into competitive tiers (Top 100, Top 20) was performed independently within each historically contested weight class, retaining results from both current and discontinued categories to maximize sample depth and historical coverage.

The methodological cornerstone of this research involved a tiered sensitivity analysis designed to isolate the phenomenon of allometric decay. Recognizing that general competitive populations contain significant variance related to suboptimal training and genetic predisposition, the dataset was stratified into three levels of competitiveness. The first tier encompassed the entire filtered population (N = 457,471; 308,743 males, 148,728 females) as a baseline for general powerlifting scaling. The second tier isolated the “Top 100” highest totals ever recorded in each historically contested weight category (N = 7,285). The third tier, defined as the “Absolute Elite,” restricted the analysis to the “Top 20” all-time unique totals per weight category (N = 1,833; 864 males, 969 females). This extreme stratification was designed to attenuate statistical noise and observe the relationship between body mass and strength at the absolute competitive zenith of the sport. Across both sexes, this procedure yielded 61 male and 71 female historically contested weight categories, reflecting the full historical evolution of weight classifications across the IPF and its affiliated federations. These categories include both current and discontinued IPF classifications, as well as a small number of non-standard decimal labels and unclassified entries (weight class recorded as blank in the OpenPowerlifting database) reflecting data entry conventions across affiliated federations. For final validation, a “Global All-Time Top 20” cohort (n = 20 per sex) was isolated, comprising the single highest-scoring athletes in history regardless of weight class to assess model behavior at the ultimate limit of human specialization. To avoid circular selection, this cohort was ranked using the empirical allometric score derived independently from the preceding Absolute Elite tier (b = 0.53 for males, b = 0.42 for females), rather than any polynomial scoring system. To confirm the robustness of the headline exponents to alternative selection criteria, a supplementary analysis was conducted selecting athletes by within-class z-score relative to the Top 20 distribution of their own weight category, a criterion involving no cross-category normalization. Under this criterion, the derived exponents were 0.534 for males and 0.426 for females, both remaining substantially below the isometric threshold of 0.67 and directionally consistent with the headline findings. To account for repeated competition entries from the same athlete, only the single highest recorded total per individual per weight class was retained for the stratified analyses, ensuring that each athlete contributes a maximum of one data point per weight category. This cohort is distinct from the Absolute Elite tier (N = 1,833), which pools the Top 20 per weight class across all historical categories.

### 2.2 Statistical analysis

All computational procedures were performed in R (version 4.3.2). The statistical relationship between total weight lifted (*S*) and athlete body mass (*M*) was modeled using the classic allometric power function (*S* = *a* × *M^b^*). To facilitate regression analysis, this relationship was transformed into a log-log format, where the natural logarithm of the total weight lifted was regressed against the natural logarithm of body mass. Within this linearized framework, the slope of the regression line represents the empirical allometric exponent (*b*). Separate regression models were constructed for male and female cohorts to account for inherent sexual differences in musculoskeletal anatomy and endocrine profiles. The statistical integrity of the derived exponents was evaluated through rigorous diagnostic protocols. The Breusch-Pagan test was employed to assess homoscedasticity, ensuring that the scaling relationship remained stable across the body mass spectrum. Furthermore, residual diagnostic plots were visually inspected to exclude systematic predictive biases at the extremes of the mass distribution. The proposed empirical allometric model was benchmarked against established international scoring systems, specifically the IPF GL formula, the DOTS coefficient, and the Wilks formula. This benchmarking approach follows validation precedents established for Olympic weightlifting by Kauhanen et al. (2002), adapted here to the powerlifting context. Mass-independence was quantified using Pearson’s product-moment correlation (*r*) between the resulting scores and body mass. To determine the statistical equivalence of the proposed model compared to the official IPF GL formula, Williams’ t-test for dependent correlations sharing one variable in common was utilized (Williams, 1959). Finally, model agreement was evaluated via Bland-Altman analysis. Thus, all scores were transformed into standardized Z-scores to neutralize the disparate mathematical scales of the scoring systems, allowing for the identification of systematic bias and 95% limits of agreement. Statistical significance was set at *α* = 0.05. One-sample *t*-tests were conducted to formally assess whether the empirically derived exponents were statistically distinguishable from the geometric ideal of 0.67, using the standard error of the regression coefficient and N-2 degrees of freedom.

To further validate the stability of the empirical exponents and mitigate the influence of extreme outliers in the micro-samples, a non-parametric bootstrapping procedure (1,000 resamples) was performed on the Absolute Elite cohort to generate 95% Confidence Intervals (CI). Additionally, a disaggregated allometric analysis was conducted on the individual powerlifts (squat, bench press, deadlift) to isolate specific regional biomechanical constraints. Finally, a rolling sensitivity analysis (from the Top 500 down to the Top 10 athletes per weight class) was executed to visually and mathematically track the continuous trajectory of the allometric decay across increasing competitive tiers. Ranking concordance between the empirical allometric model and the IPF GL formula was assessed using within-class Spearman’s ρ and mean absolute rank displacement, computed across all historically contested weight classes within the Absolute Elite cohort. To confirm the robustness of the headline Absolute Elite exponents to the exclusion of non-standard weight class strata, a sensitivity analysis was conducted excluding all unclassified entries and weight class strata containing only a single athlete.

## 3 Results

### 3.1 Participant characteristics

The final dataset representing the general competitive powerlifting population comprised 457,471 athletes. Stratification of these data by competitive tier yielded 7,285 elite lifters for the “Top 100” cohort. The most restrictive tier, representing the “Absolute Elite” (the top 20 all-time highest totals per official and historical weight class), consisted of 1,833 individuals. **Table 1** summarizes detailed descriptive statistics, including age, body mass, absolute total weight lifted, and corresponding IPF GL scores, presented as means ± standard deviations (SD), across all three competitive tiers.

**Table 1:** Descriptive characteristics of the participants across competitive tiers.

Competitive tier	Sex	N	Age (years)	Body mass (kg)	Total (kg)	IPF GL score
Total Population	F	148,728	26.4 ± 4.2	69.8 ± 16.5	328.3 ± 71.7	69.7 ± 13.6
	M	308,743	25.7 ± 4.1	91.1 ± 18.9	581.3 ± 110.7	77.4 ± 12.1
Top 100 Elite	F	3,708	27.1 ± 4.3	75.6 ± 26.3	424.3 ± 106.2	88.9 ± 17.6
	M	3,577	26.5 ± 4.2	96.0 ± 31.0	709.2 ± 176.3	93.0 ± 17.1
Top 20 Absolute Elite	F	969	27.3 ± 4.3	77.5 ± 28.0	444.2 ± 120.0	92.3 ± 20.8
	M	864	27.2 ± 4.3	99.3 ± 32.1	756.0 ± 177.3	97.9 ± 16.8

Sex, Female (F) and Male (M); N, number of observations.

To contextualize these findings within contemporary competition standards, a comprehensive breakdown of participant distribution and average performance metrics restricted strictly to current official IPF weight classes is provided in the **Supplementary Table 1**. This progressive narrowing of the dataset successfully isolated the absolute upper echelons of human strength, establishing the necessary foundation for the subsequent analysis of allometric scaling limits.

### 3.2 Allometric scaling and the “decay” phenomenon

**Figure 1:**
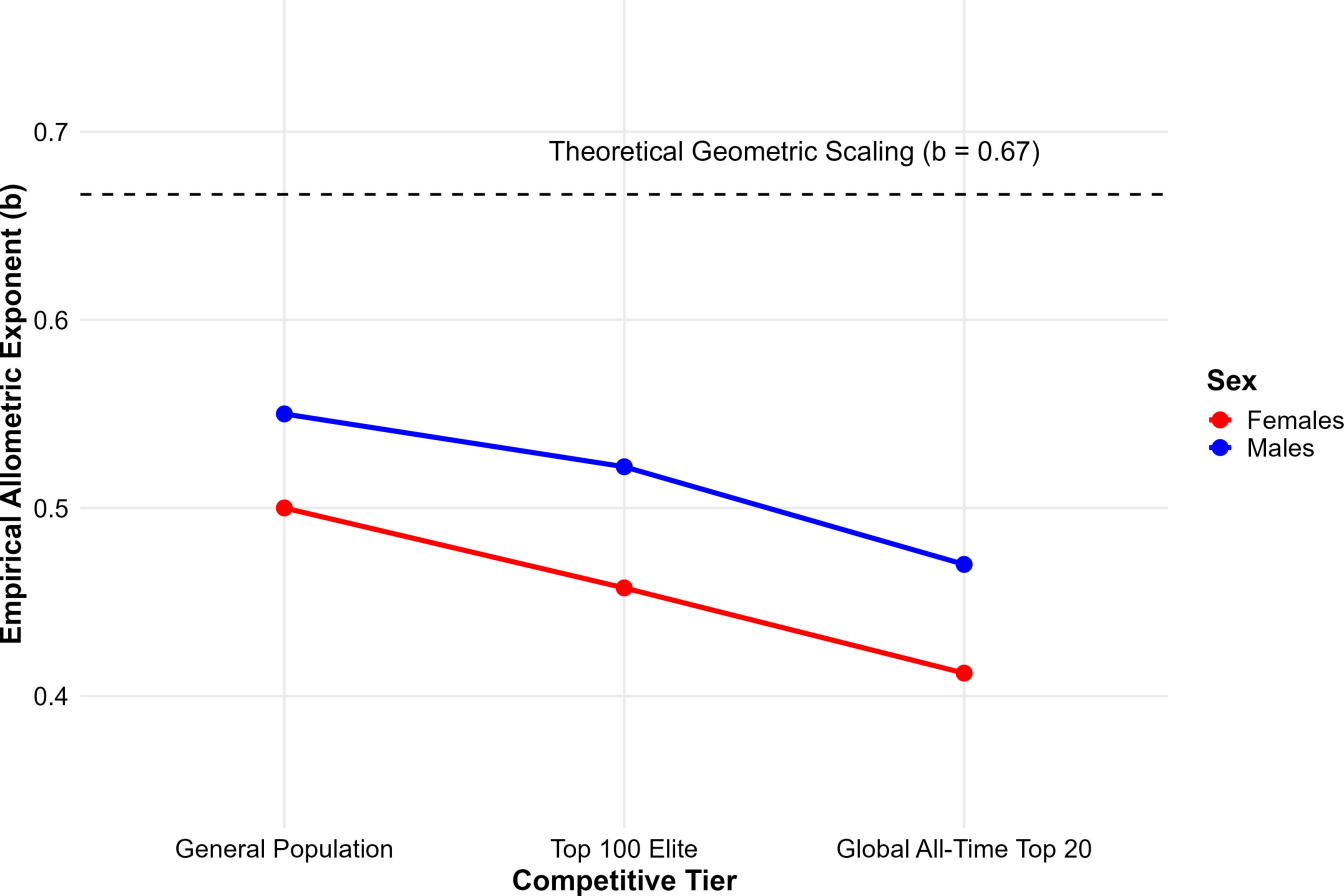
The phenomenon of allometric decay. The empirical scaling exponent (b) for both sexes systematically decreases as performance levels approach the observed competitive performance limit.

The application of log-log regression models across the stratified competitive tiers yielded a confirmation of the primary hypothesis, revealing a systematic and progressive downward shift in the allometric exponent as sample density shifted from the total population toward the absolute elite. This phenomenon, classified as allometric decay, illustrates a clear departure from theoretical isometric scaling as performance approaches observed competitive limits (**Table 2**; **Figure 1**).

**Table 2:** Evolution of the allometric exponent (b) across competitive tiers.

Competitive tier	N (males)	N (female)	Male b (SE)	Male R²	Female b (SE)	Female R²
Theoretical (Isometric)			0.667 (-)	–	0.667 (-)	–
Total Population	308,530	148,685	0.550 (0.002)	0.30	0.500 (0.002)	0.18
Top 100 Elite	3,576	3,707	0.522 (0.011)	0.45	0.458 (0.012)	0.29
Absolute Elite (Top 20 per class)	864	969	0.530 (0.021)	0.42	0.420 (0.025)	0.23

SE, Standard error; *p < 0.05. N reflects athletes with complete data used in the regression analysis.

Within the baseline tier representing the general competitive population, the male allometric exponent was calculated at 0.55, while the female exponent settled at 0.50. These values are notably lower than the geometric ideal of 0.67, confirming that even at sub-elite levels, strength does not scale proportionally with increasing body volume.

At “Top 100” tier, the exponents exhibited further attenuation, reaching 0.52 for males and 0.45 for females. The most profound revelation occurred during the final validation phase utilizing the “Global All-Time Top 20” cohort, representing the absolute zenith of human physiological specialization regardless of weight class. Within this ultimate micro-sample, the allometric score derived from the Absolute Elite tier (b = 0.53 for males, b = 0.42 for females) was used to rank athletes cross-category. The resulting Global All-Time Top 20 cohort exhibited optimal mass-independence at b = 0.47 for males and b = 0.41 for females, values that minimize residual body mass correlation in this micro-sample and are consistent with the decay trajectory observed across tiers.

Diagnostic evaluations confirmed the statistical robustness of these findings; the Breusch-Pagan test, applied to the Global All-Time Top 20 cohort, yielded *p*-values of 0.54 and 0.27 for the elite male and female models, respectively. These results did not detect heteroscedasticity at *α* = 0.05 for either the male or female models. It should be noted that the statistical power of the Breusch-Pagan test is limited at n = 20.

### 3.3 Comparative validation and statistical equivalence

**Figure 2:**
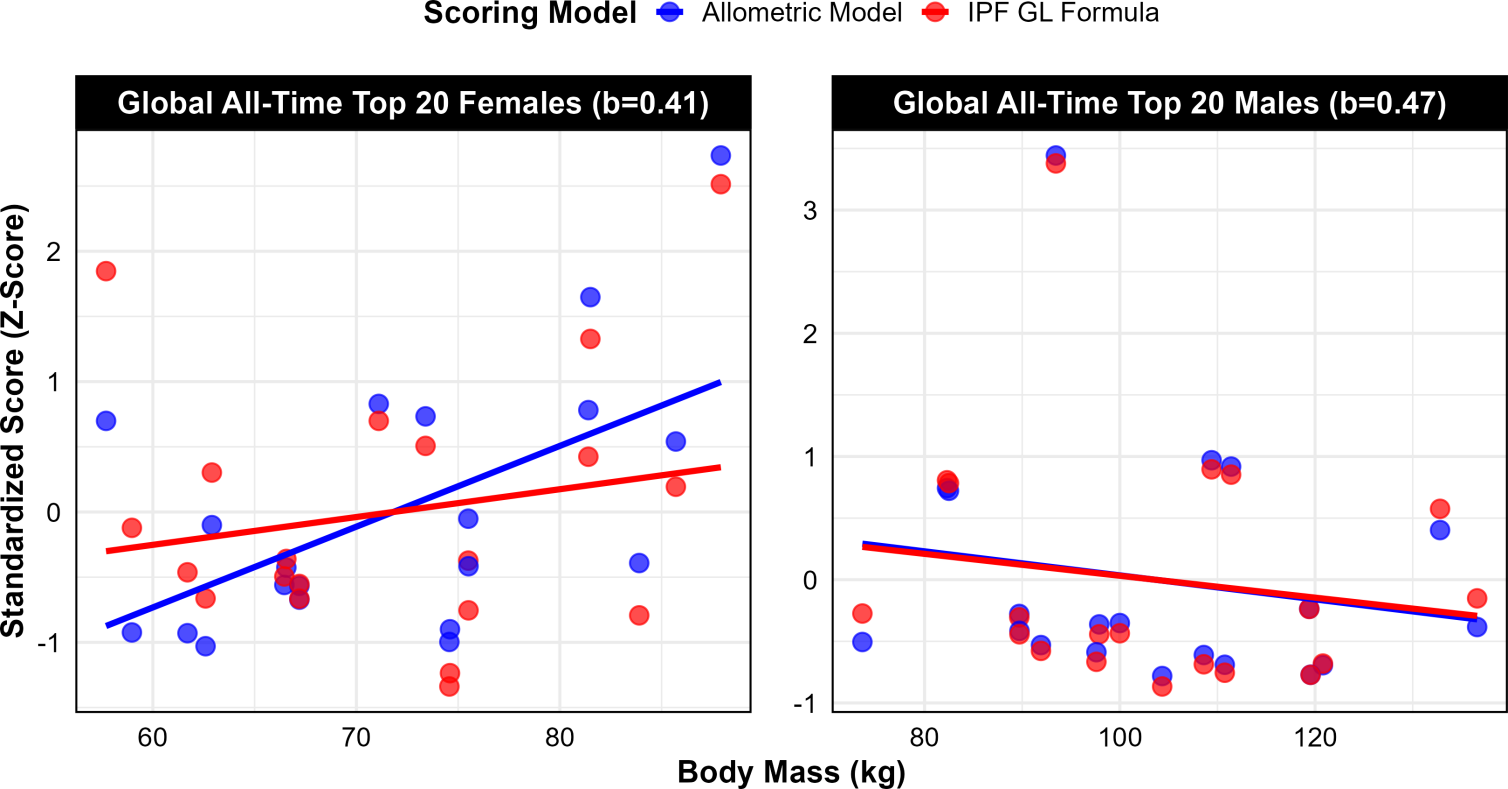
Comparison of mass-bias between empirical allometric models and the IPF GL formula (Global All-Time Top 20 cohort, N = 20 per sex).

The benchmarking of the proposed empirical allometric models against established international standards revealed that a mean power function can achieve levels of mass-independence comparable to highly complex polynomial algorithms (**Figure 2**). Analysis of the absolute male cohort indicated that while the legacy Wilks formula exhibited the highest residual bias (*r* = 0.103), the contemporary DOTS and IPF GL formulas achieved higher levels of equity (*r* = 0.064 and *r* = 0.089, respectively). The proposed male allometric model (*b* = 0.47) yielded a correlation of 0.091, which Williams’ t-test confirmed to be statistically equivalent to the official IPF GL formula (*t* = -0.72, *p* = 0.47, Cohen’s *q* = 0.001). Similarly, for the female cohort, the derived exponent (*b* = 0.41) effectively neutralized mass-bias in the broader elite categories. When subjected to an extreme stress-test against the global all-time Top 20 females, the allometric model exhibited a residual bias of *r* = 0.56, compared to *r* = 0.19 for the IPF GL formula. Although Williams’ t-test indicated a significant difference in this specific micro-sample (*t* = -9.75, *p* < 0.001, Cohen’s *q* = 0.439), this represents a substantive limitation of the proposed allometric approach at the extreme micro-sample level for female athletes. The divergence is structurally driven by the concentration of the Global All-Time Top 20 females in lightweight categories, a distributional feature that favors polynomial curve-fitting over a fixed-exponent power function. This limitation does not extend to the broader Absolute Elite tier, where the allometric model performs consistently across the full weight class spectrum.

### 3.4 Agreement analysis between scoring systems

**Figure 3:**
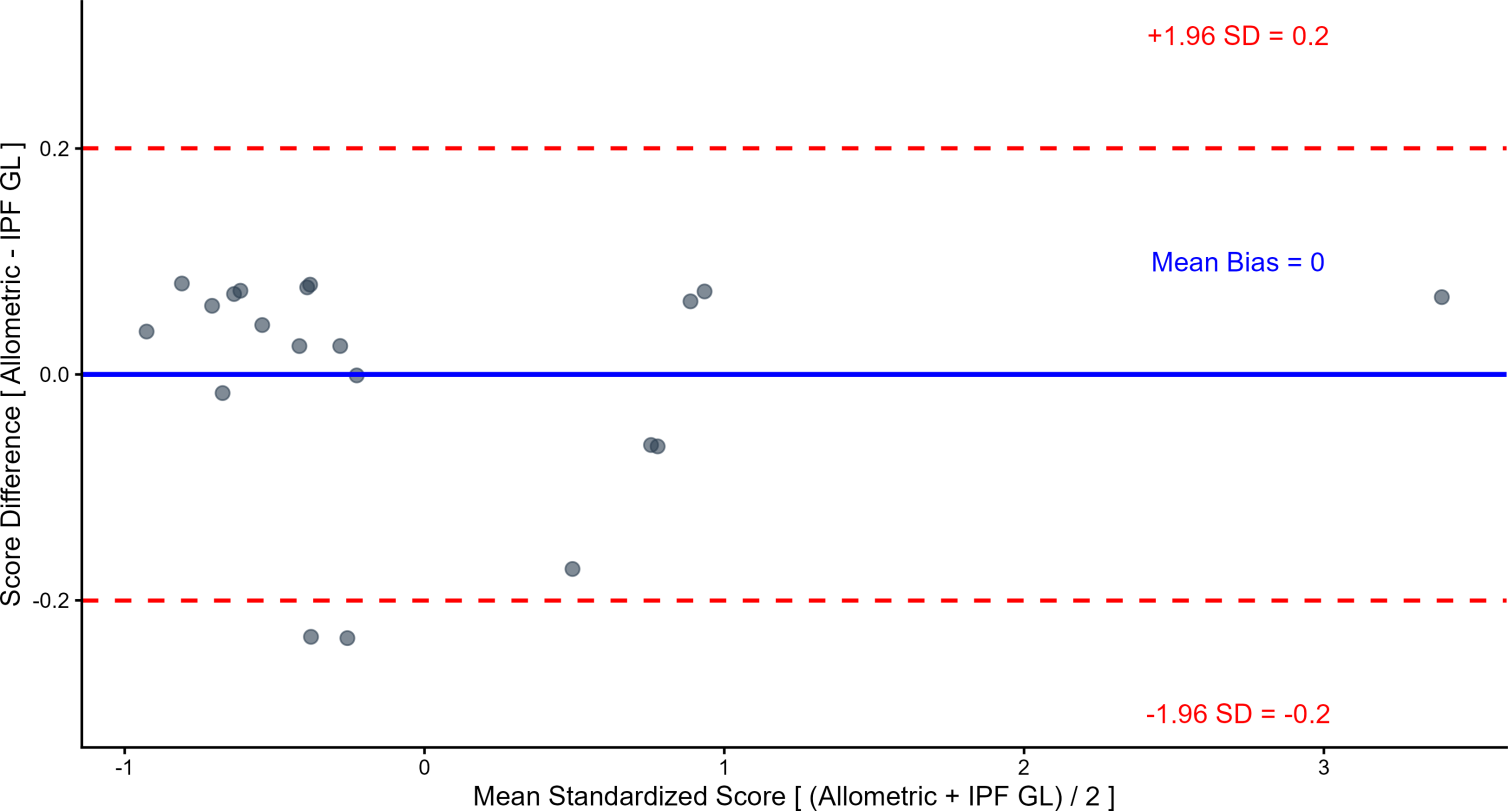
Bland-Altman agreement plot between standardized scores of the empirical allometric model and the IPF GL formula for the male Global All-Time Top 20 cohort (N = 20).

**Figure 4:**
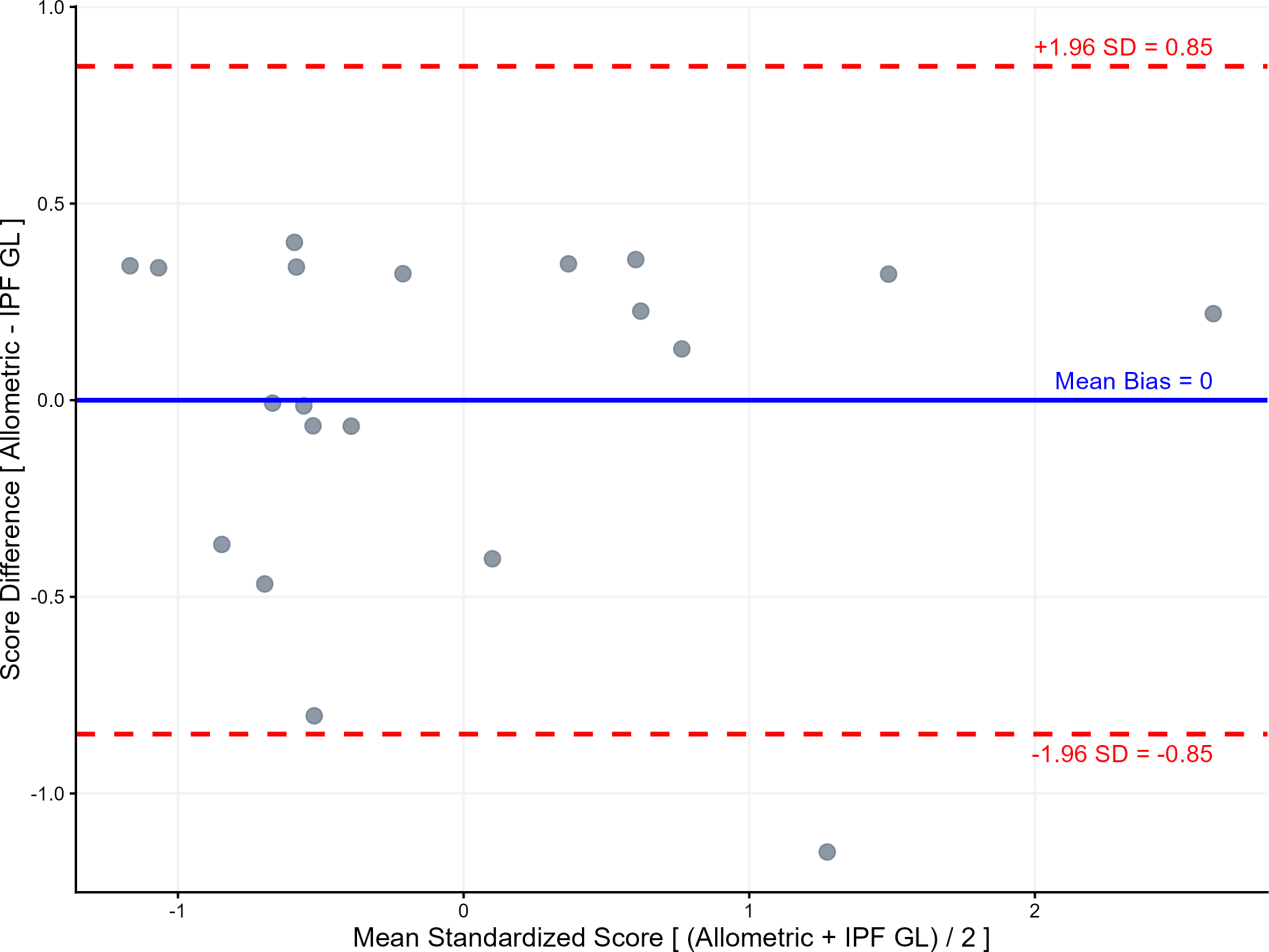
Bland-Altman agreement plot between standardized scores of the empirical allometric model and the IPF GL formula for the female Global All-Time Top 20 cohort (N = 20).

The agreement between the proposed models and the IPF GL system was visualized via Bland-Altman plots of standardized Z-scores (**Figures 3**, **4**). The analysis for both sexes demonstrated a mean bias of 0.00 and narrow 95% limits of agreement (± 0.20 for males; ± 0.85 for females), with no evidence of proportional bias. The regression slope of difference on mean was 0.000 for males (95% CI [-0.051, 0.051]) and 0.000 for females (95% CI [-0.220, 0.220]), formally confirming the absence of proportional bias in both models. These findings confirm that the empirical allometric approach and the complex IPF GL algorithm provide nearly identical rankings for elite athletes. This suggests that the disruptive impact of generational outliers, such as those observed in the 90 kg male and various female lightweight classes, affects both mathematical architectures equally, further reinforcing the biological validity of the allometric power function at the limits of human strength.

### 3.5 Robustness, sensitivity, and disaggregated analysis

**Figure 5:**
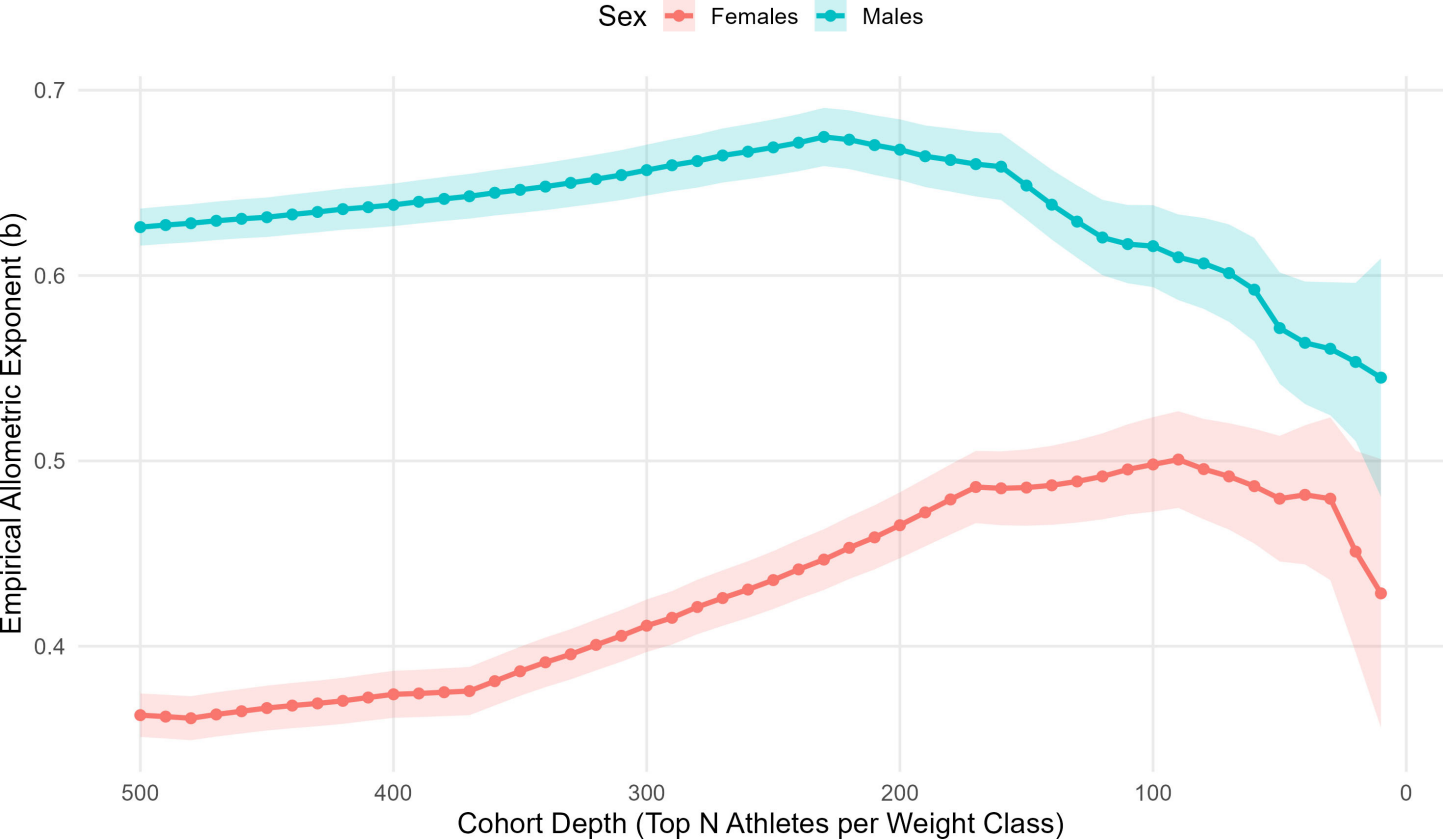
Evidence of a biphasic scaling trajectory in elite powerlifters.

To confirm the continuous nature of the allometric decay, a rolling sensitivity analysis was performed. As the cohort density was progressively restricted from the Top 500 down to the absolute Top 10 athletes per weight class, the empirical scaling exponents for both sexes exhibited a biphasic trajectory (**Figure 5**). In the broader cohort range (approximately Top 500 to Top 200), the exponent rises progressively, reflecting improved representation of heavier weight categories as sub-elite athletes are excluded. In the narrower range (approximately Top 200 to Top 10), the exponent declines toward the values observed in the Absolute Elite tier, consistent with the observed scaling constraints characterizing the upper frontier of competitive performance. This biphasic pattern is not inconsistent with the allometric decay hypothesis; rather, it reveals two competing forces: a selection effect operating at intermediate cohort depths, and an observed performance constraint emerging at the absolute elite level.

Furthermore, a non-parametric bootstrapping procedure (1,000 resamples) on the Absolute Elite cohort (Top 20 per weight class; N = 864 males, N = 969 females) validated the stability of the derived exponents for this tier. The male Absolute Elite cohort yielded a corrected 95% bootstrap CI of [0.483, 0.584] around the point estimate b = 0.53, remaining entirely below the theoretical geometric constant of 0.67. The female Absolute Elite cohort yielded a 95% bootstrap CI of [0.370, 0.470] around the point estimate b = 0.42, likewise remaining entirely below the isometric threshold. One-sample t-tests performed on the Absolute Elite cohort confirmed that both tier-specific exponents are statistically distinguishable from the geometric ideal of 0.67 (males: b = 0.53, t = -6.591, p < 0.001; females: b = 0.42, t = -10.086, p < 0.001). At the terminal validation level, the Global All-Time Top 20 cohort (n = 20 per sex), comprising the highest-scoring athletes regardless of weight class, selected using the Absolute Elite exponents, exhibited optimal mass-independence at b = 0.47 for males and b = 0.41 for females, representing the empirically optimized values at the absolute frontier of competitive performance and consistent with the decay trajectory established across the three formal regression tiers. A further sensitivity analysis excluding all unclassified entries and weight class strata containing only a single athlete from the Absolute Elite tier yielded exponents of b = 0.495 (SE = 0.021) for males and b = 0.406 (SE = 0.025) for females, both remaining substantially below the isometric threshold of 0.67 and fully consistent with the headline findings.

To isolate the biomechanical drivers of this variance, a disaggregated analysis of the individual powerlifts was conducted (**Table 3**). For males, the attenuation was most prominent in the bench press (*b* = 0.345) and deadlift (b = 0.394), while the squat exhibited higher resilience to mass growth (*b* = 0.515). Crucially, in the female cohort, the bench press demonstrated a systematically depressed exponent (*b* = 0.424), heavily weighing down the overall Total, whereas the female deadlift maintained highly efficient scaling (*b* = 0.691). These patterns are visualized in **Supplementary Figure 1**, which presents separate allometric scaling fits for each lift by sex.

**Table 3:** Disaggregated allometric exponents for the Global All-Time Top 20 cohort (N = 20 per sex), comprising the highest-scoring athletes regardless of weight class.

Lifts	Male b	Female b
Squat	0.515	0.542
Bench Press	0.345	0.424
Deadlift	0.394	0.691

To provide applied validation of the proposed allometric formula, a supplementary ranking comparison between the empirical allometric model and the IPF GL was conducted across all historically contested weight classes within the Absolute Elite cohort (**Supplementary Table 2**). The analysis revealed mean within-class Spearman ρ of 0.995 for males and 0.976 for females, with mean absolute rank displacements of 0.146 and 0.435 positions respectively. These findings confirm near-identical ranking concordance for males and very high concordance for females, while acknowledging greater within-class variability in female heavy and super-heavy categories, consistent with the female divergence reported elsewhere in the manuscript.

## 4 Discussion

The primary aim of this study was to derive empirical allometric scaling exponents for elite powerlifters and evaluate their efficacy against current polynomial algorithms. The findings of this large-scale analysis refute the presumption that geometric isometry can be accurately applied to elite human strength performance. The significant gulf between the theoretical square-cube constant of 0.67 and the empirical observations of 0.47 for males and 0.41 for females is consistent with biological constraints governing muscular hypertrophy and force production, though direct causal inference is limited by the observational nature of competition data. The physiological mechanisms discussed in the following paragraphs should therefore be interpreted as theoretically plausible hypotheses consistent with the observed scaling patterns, rather than as causally established explanations. This finding suggests that as an athlete approaches their observed performance ceiling, the relationship between mass and power undergoes a notable shift, moving away from Euclidean geometry and toward a more restrictive biological scaling law (Åstrand et al., 2003; Jaric, 2002). A methodologically parallel investigation in Olympic weightlifting by Huebner and Perperoglou (2020), using competitive records and a sex-stratified approach, reported convergent findings regarding the inadequacy of universal scaling constants across body mass categories. Notably, the male elite exponent derived in the present study (*b* = 0.47) is numerically identical to that reported by Batterham and George (1997) for elite Olympic weightlifters at the 1995 World Championships (*b* = 0.47, 95% CI 0.43-0.51). This convergence is, in our view, more than a numerical coincidence. Both sports share a common physiological substrate of maximal force production, lending plausibility to this empirical correspondence. This convergence should be interpreted as a corroborating empirical observation rather than evidence of a universal law, particularly given that Batterham and George (1997) themselves concluded that allometric modeling does not determine a dimensionless power-function ratio, that their b ≈ 0.47 estimate was mass-dependent and sensitive to the inclusion of the heaviest lifters, and that the female exponent of 0.41 diverges meaningfully from this value.

The sources driving this allometric decay are multifactorial, rooted deeply in body composition and functional biomechanics. As athletes undergo heavyweight and super-heavyweight categories, they inevitably confront the upper boundaries of the human Fat-Free Mass Index (FFMI) (Kouri et al., 1995). To continue increasing total body mass beyond these natural lean-tissue limits, athletes rely on disproportionately higher ratios of non-contractile adipose tissue. This tissue effectively acts as metabolic and mechanical ballast, increasing the total inertia and mass that must be displaced during the execution of a lift, while contributing negligibly to active force production. This ‘attenuation’ of contractile density explains why the addition of total body mass yields diminishing returns in absolute strength.

Furthermore, the extreme physical dimensions of elite super-heavyweight athletes introduce significant mechanical disadvantages. Increased girth of the torso, thighs, and upper arms may result in ‘soft tissue interference’, which can prematurely truncate the optimal joint range of motion and negatively affect joint mechanics in the squat and deadlift, a mechanical phenomenon theorized in super-heavyweight athletes though not directly quantified in this population. For instance, excessive abdominal or thigh girth may force a wider, less mechanically efficient stance or alter the lifter’s center of mass, increasing the torque required for stance and lifting at the hips and lower back. Consequently, the metabolic and mechanical cost of moving each additional kilogram of body mass increases exponentially, leading directly to the reduced allometric exponents observed in the absolute elite cohorts.

Equally significant is the profound sexual difference revealed by the scaling exponents. The finding that the female global exponent decays to 0.41, substantially lower than the male 0.47, initially suggests earlier diminishing returns on mass growth. However, the disaggregated analysis provides a crucial biomechanical explanation, isolating the limitation to upper-body hypertrophy. The bench press exhibited the most systematic decay across the sport, dropping to 0.35 for males and 0.42 for females. For female athletes, this restriction is particularly decisive. While their lower-body and posterior chain mechanics maintain robust scaling (with the female squat and deadlift yielding exponents of 0.54 and 0.69, respectively), the severe upper-body attenuation functions as a biological bottleneck, depressing the overall total exponent. The contrast between the high scaling efficiency of the female deadlift and the progressive decay of the bench press elucidates the confidence intervals observed in the female bootstrap analysis. It should be noted, however, that this biomechanical complexity also translates into a practical limitation of the proposed fixed-exponent model for female athletes at the micro-sample level. When applied to the Global All-Time Top 20 females, a cohort structurally dominated by lightweight athletes, the allometric model yields a residual mass-bias substantially higher than the IPF GL formula (*r* = 0.56 vs *r* = 0.19). This limitation should be considered when evaluating the practical applicability of the proposed formula for female cross-category comparisons at the absolute elite level. This notion aligns with evidence that androgen receptors are expressed in human skeletal muscle and upregulated by androgen treatment (Sinha-Hikim et al., 2004), and that sex differences in regional skeletal muscle mass distribution result in proportionally lower upper-body muscle mass in females relative to males (Janssen et al., 2000). Sex-specific allometric differences in strength sports have also been documented by Vanderburgh et al. (1997), whose findings are consistent with the divergent male and female exponents reported here. The employment of a universal, non-sex-specific scoring formula is therefore inherently biased, as it fails to account for the biological boundaries where female strength resists mass-to-power conversion at a significantly steeper rate of decay. The phenomenon of allometric decay exposes the primary flaw in utilizing over-parameterized polynomial algorithms, such as the IPF GL formula, to govern competitive strength sports (Vanderburgh and Batterham, 1999). Polynomials are essentially descriptive “curve-fitting” tools that are highly susceptible to dataset-specific overfitting based on the specific density of the historical data provided. It should be acknowledged that these polynomial systems were deliberately designed as competitive ranking tools rather than physiological models, and they perform this function effectively within their intended scope. If a federation’s database contains a high density of sub-maximal heavyweights, the polynomial curve will artificially descend to accommodate them, creating mathematical artifacts that may not generalize beyond the specific population from which the polynomial was derived. In contrast, empirical allometry recognizes that a 150-kg human is biologically incapable of maintaining the same muscle-to-mass ratio as a 75-kg human (Batterham and George, 1997; Jaric, 2002). By replacing impervious, multi-parameter algorithms with a parsimonious allometric power function, the sport moves from adjusting mathematics to fit the population to measuring the athlete against the empirically observed performance limits of the sport.

A notable limitation of this study is its strict confinement to drug-tested populations. The administration of exogenous anabolic-androgenic steroids fundamentally alters the physiological ceiling of the FFMI, allowing athletes to increase contractile tissue far beyond natural limits (Kouri et al., 1995). An identical analysis performed on federations without drug testing protocols may yield different scaling exponents; however, the direction and magnitude of any such difference cannot be established from the current data and should be treated as a hypothesis for future research. Importantly, untested federation is a regulatory descriptor and does not imply that participants use performance-enhancing substances, as many athletes compete across both tested and untested competitions within the same calendar year (Hartgens and Kuipers, 2004). Future research should prioritize a comparative analysis between tested and untested databases to precisely quantify the impact of performance-enhancing drugs on human scaling laws.

## 5 Conclusion

This study provides strong empirical evidence that the observed scaling of competitive strength performance is a dynamic, decaying parameter that is highly sensitive to both the athlete’s proximity to their observed competitive potential and their biological sex. The finding that the absolute limit of male strength scales at a power of 0.47, and female strength at 0.41, renders the theoretical square-cube law and over-parameterized polynomial formulas obsolete for elite competition. We advocate for the global adoption of Sex-Specific Empirical Allometry. By applying a straightforward power function utilizing exponents of 0.5 for men and 0.4 for women as biologically grounded, mathematically elegant heuristics, the strength and conditioning community can establish a definitive, transparent, and undeniably fair standard for determining the ultimate limits of human physical power.

From a highly practical standpoint, the implications of these findings are twofold. In general strength and conditioning, the simplified exponents of 0.5 for men and 0.4 for women provide coaches with a rapid, intuitive metric to track an athlete’s relative strength progress across different training blocks, independently of body mass fluctuations. In competitive powerlifting, adopting this empirical allometry offers a transparent and biologically equitable method for cross-category rankings and “Best Lifter” awards. The applied ranking comparison presented in **Supplementary Table 2** further confirms that no weight class is systematically advantaged or disadvantaged under the proposed formula relative to the IPF GL, supporting its practical viability as a replacement scoring system. By replacing opaque algorithmic “black boxes” with a straightforward power function, federations can finally ensure that heavyweight athletes are not mathematically penalized, allowing competitors of all sizes to be evaluated on a genuinely level playing field. 

## Data Availability

Publicly available datasets were analyzed in this study. This data can be found here: https://openpowerlifting.gitlab.io/opl-csv/.
