# Supplementary material for: An allometric large-scale analysis of the observed performance limits and sex differences in elite powerlifting
Source: Front Physiol. 2026 Jun 24;17:1847605. doi: 10.3389/fphys.2026.1847605 (PMC13341520; doi:10.3389/fphys.2026.1847605)
Supplement: Supplementary file 1 [file DataSheet1.pdf]

Supplementary Material

Supplementary Table S1. Participant stratification by official IPF weight class across tiers.

Competitive Tier	Weight Class (kg)	Male N	Male Avg Total (kg)	Female N	Female Avg Total (kg)
1. General Population	47	-	-	2,674	266.4
	52	-	-	9,606	280.8
	57	-	-	11,229	305.8
	59	3,655	435.2	-	-
	63	-	-	15,878	320.0
	66	9,866	481.2	-	-
	69	-	-	8,049	352.0
	72	-	-	9,172	325.0
	74	22,322	519.2	-	-
	76	-	-	6,346	367.9
	83	34,980	556.3	-	-
	84	-	-	9,486	357.4
	84+	-	-	7,170	382.1
	93	37,216	590.5	-	-
	105	27,828	627.5	-	-
	120	14,001	661.0	-	-
	120+	7,424	698.4	-	-
2. Top 100 Elite	47	-	-	100	359.3
	52	-	-	100	412.0
	57	-	-	100	450.6
	59	100	572.9	-	-
	63	-	-	100	481.9
	66	100	661.1	-	-
	69	-	-	100	503.6
	72	-	-	100	475.2
	74	100	733.1	-	-
	76	-	-	100	523.6
	83	100	795.0	-	-
	84	-	-	100	534.1
	84+	-	-	100	574.7
	93	100	841.3	-	-
	105	100	876.6	-	-
	120	100	907.7	-	-
	120+	100	952.5	-	-
3. Top 20 Absolute Elite	47	-	-	20	399.6
	52	-	-	20	443.9
	57	-	-	20	488.1

Competitive Tier	Weight Class (kg)	Male N	Male Avg Total (kg)	Female N	Female Avg Total (kg)
	59	20	614.7	-	-
	63	-	-	20	525.0
	66	20	704.9	-	-
	69	-	-	20	554.1
	72	-	-	20	517.0
	74	20	775.5	-	-
	76	-	-	20	568.6
	83	20	838.7	-	-
	84	-	-	20	583.0
	84+	-	-	20	663.5
	93	20	889.2	-	-
	105	20	922.1	-	-
	120	20	957.6	-	-
	120+	20	1015.0	-	-

Supplementary Table S2. Within-class ranking concordance between IPF GL and empirical allometric formula by weight class (Absolute Elite Tier).

Sex	Weight Class (kg)	N	Spearman ρ	Mean Absolute Δ Rank	SD Δ Rank
Male	40	1		0.00	
	52	20	1.0000000	0.00	0.00
	53	20	1.0000000	0.00	0.00
	56	20	0.9984940	0.10	0.32
	59	20	0.9984962	0.10	0.32
	60	20	0.9984940	0.10	0.32
	62	20	0.9969925	0.20	0.46
	66	20	0.9966128	0.25	0.51
	67.5	20	1.0000000	0.00	0.00
	69	20	1.0000000	0.00	0.00
	70	1		0.00	
	74	20	0.9984962	0.10	0.32
	75	20	0.9984951	0.10	0.32
	77	20	0.9939850	0.30	0.65
	79.3	6	1.0000000	0.00	0.00
	79.5	3	1.0000000	0.00	0.00
	80	13	1.0000000	0.00	0.00

Sex	Weight Class (kg)	N	Spearman ρ	Mean Absolute Δ Rank	SD Δ Rank
	82	5	1.0000000	0.00	0.00
	82.5	20	1.0000000	0.00	0.00
	83	20	0.9984962	0.10	0.32
	85	20	1.0000000	0.00	0.00
	90	20	0.9984962	0.10	0.32
	90.5	1		0.00	
	90.7	3	1.0000000	0.00	0.00
	93	20	1.0000000	0.00	0.00
	94	20	0.9954887	0.20	0.56
	95	20	1.0000000	0.00	0.00
	100	20	1.0000000	0.00	0.00
	102	13	1.0000000	0.00	0.00
	104.7	16	1.0000000	0.00	0.00
	105	20	1.0000000	0.00	0.00
	109	2	1.0000000	0.00	0.00
	110	20	0.9984962	0.10	0.32
	113.5	1		0.00	
	114	1		0.00	
	115	5	1.0000000	0.00	0.00

Sex	Weight Class (kg)	N	Spearman ρ	Mean Absolute Δ Rank	SD Δ Rank
	118	20	0.9984962	0.10	0.32
	120	20	0.9996240	0.05	0.22
	125	20	0.9954887	0.30	0.56
	132	1		0.00	
	136	1		0.00	
	140	20	0.9984962	0.10	0.32
	145	20	0.9879609	0.50	0.92
	Unclassified	20	0.9909774	0.50	0.79
	+	9	1.0000000	0.00	0.00
	100+	20	0.9954887	0.30	0.56
	105+	20	0.9894737	0.40	0.86
	110+	20	0.9729323	0.80	1.38
	113.5+	2	1.0000000	0.00	0.00
	120+	20	0.9819549	0.70	1.12
	120.2+	1		0.00	
	125+	20	0.9729323	1.00	1.38
	140+	20	0.9263158	1.50	2.27
	145+	9	0.9833333	0.22	0.50
	69+	3	1.0000000	0.00	0.00
	82.5+	6	1.0000000	0.00	0.00

Sex	Weight Class (kg)	N	Spearman ρ	Mean Absolute Δ Rank	SD Δ Rank
	83+	20	1.0000000	0.00	0.00
	85+	20	0.9984962	0.10	0.32
	90+	20	0.9939850	0.30	0.65
	93+	20	0.9981197	0.15	0.39
Female	40	1		0.00	
	43	20	0.9834586	0.70	1.08
	44	20	0.9774436	0.80	1.26
	47	20	0.9924812	0.40	0.73
	47.5	16	0.9970588	0.12	0.37
	48	20	0.9864662	0.60	0.97
	50	3	1.0000000	0.00	0.00
	50.5	20	0.9894737	0.50	0.86
	52	20	0.9864662	0.50	0.97
	53	20	0.9518797	1.10	1.84
	54.4	1		0.00	
	55	3	1.0000000	0.00	0.00
	55.5	20	0.9864662	0.70	0.97
	56	20	0.9969925	0.20	0.46
	57	20	0.9714286	1.00	1.41

Sex	Weight Class (kg)	N	Spearman ρ	Mean Absolute Δ Rank	SD Δ Rank
	58	20	0.9819549	0.60	1.12
	58.5	20	0.9984962	0.10	0.32
	59	6	1.0000000	0.00	0.00
	60	20	0.9879699	0.50	0.92
	61	2	1.0000000	0.00	0.00
	63	20	0.9864662	0.50	0.97
	63.5	7	1.0000000	0.00	0.00
	64	20	0.9879699	0.60	0.92
	65	20	0.9744361	0.90	1.34
	66	1		0.00	
	67.5	20	0.9729323	1.10	1.38
	69	20	0.9984962	0.10	0.32
	70	20	0.9924812	0.30	0.73
	72	20	0.9939850	0.30	0.65
	72.5	3	1.0000000	0.00	0.00
	75	20	0.9984962	0.10	0.32
	76	20	0.9627680	0.95	1.67
	80	20	0.9984962	0.10	0.32
	81.5	13	1.0000000	0.00	0.00
	81.6	5	1.0000000	0.00	0.00

Sex	Weight Class (kg)	N	Spearman ρ	Mean Absolute Δ Rank	SD Δ Rank
	82	7	1.0000000	0.00	0.00
	82.5	20	1.0000000	0.00	0.00
	83	1		0.00	
	84	20	0.9969925	0.20	0.46
	90	20	0.9939804	0.30	0.65
	90.5	1		0.00	
	90.7	1		0.00	
	100	20	0.9969925	0.20	0.46
	105	1		0.00	
	110	20	0.9939850	0.30	0.65
	118	1		0.00	
	125	20	0.9774436	0.80	1.26
	140	20	0.9939850	0.40	0.65
	Unclassified	20	0.8796992	2.10	2.90
	+	10	0.9878788	0.20	0.47
	100+	20	0.6646617	3.40	4.84
	110+	20	0.8330827	2.30	3.42
	140+	15	0.9857143	0.53	0.76
	56+	7	1.0000000	0.00	0.00

Sex	Weight Class (kg)	N	Spearman ρ	Mean Absolute Δ Rank	SD Δ Rank
	57+	20	0.9248120	1.70	2.29
	60+	20	0.9984962	0.10	0.32
	63+	20	0.9684211	0.80	1.49
	64+	1		0.00	
	67.5+	17	0.9975490	0.12	0.35
	69+	20	0.9729323	0.70	1.38
	70+	2	1.0000000	0.00	0.00
	72+	12	1.0000000	0.00	0.00
	72.5+	1		0.00	
	75+	20	0.9984962	0.10	0.32
	76+	20	0.9984962	0.10	0.32
	80+	1		0.00	
	82+	3	1.0000000	0.00	0.00
	82.5+	6	0.9428571	0.33	0.63
	84+	20	0.9293233	1.70	2.22
	90+	20	0.8887218	1.70	2.79
	90.7+	1		0.00	

Unclassified: athletes with no weight class recorded in the OpenPowerlifting database, retained based on elite-level performance totals.

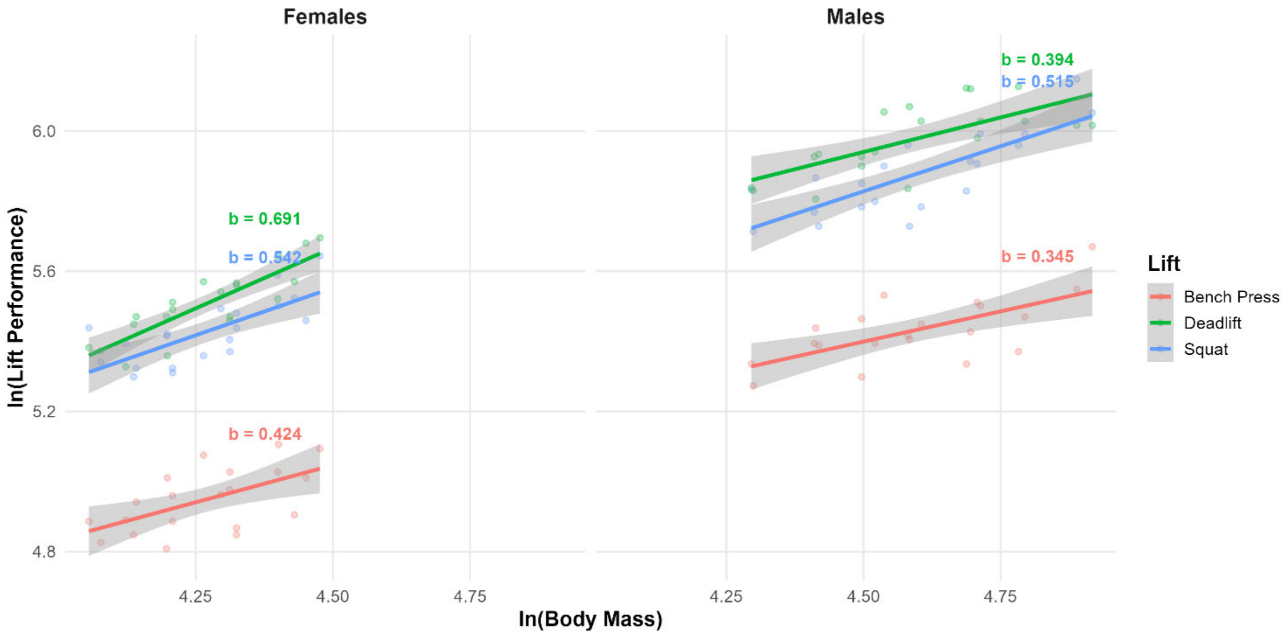

Supplementary Figure S1. Separate log-log regression fits for the bench press, deadlift, and squat for both female and male cohorts within the Global All-Time Top 20 cohort (N = 20 per sex).
